# Supplementary material for: Tuning charge and correlation effects for a single molecule on a graphene device
Source: Nat Commun. 2016 Nov 25;7:13553. doi: 10.1038/ncomms13553 (PMC5133630; doi:10.1038/ncomms13553)
Supplement: Supplementary Information — Supplementary Figures 1-11, Supplementary Notes 1-4 and Supplementary References [file ncomms13553-s1.pdf]

Supplementary Figures

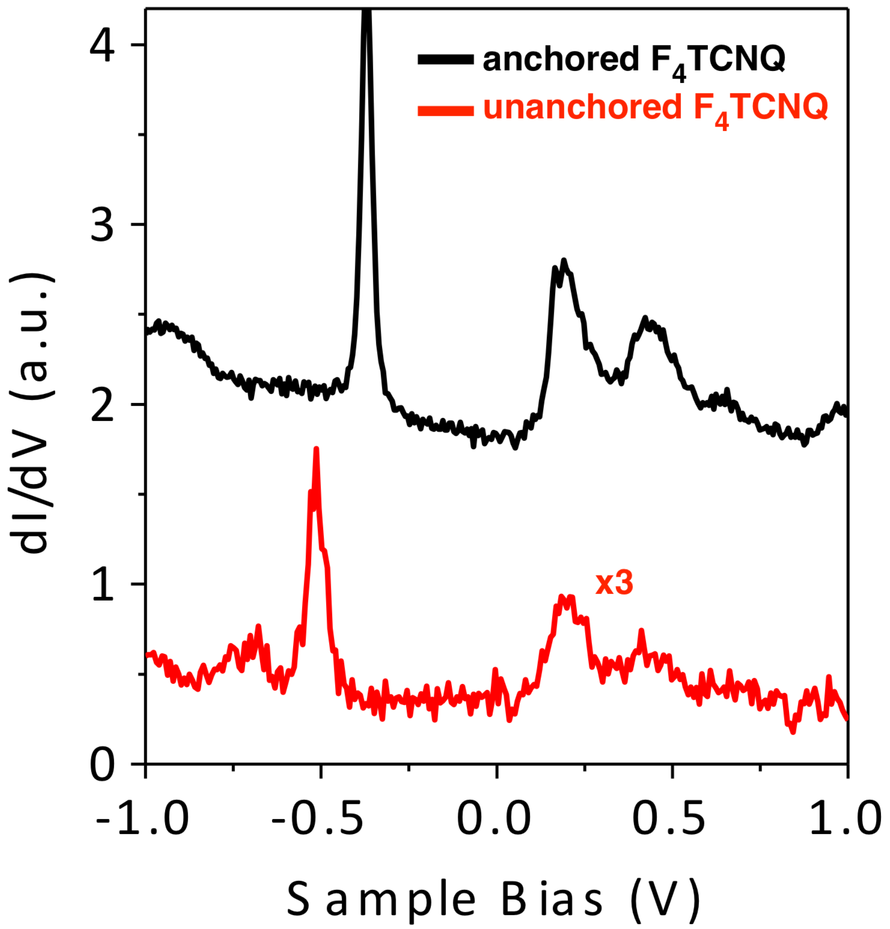

Supplementary Figure 1. Spectroscopy of anchored (to PCDA) and unanchored F_4TCNQ molecules on graphene/BN is qualitatively similar. However, for unanchored molecules the initial tunneling current is chosen to be lower to avoid moving the molecule during measurement, resulting in lower signal-to-noise ratio and shifted charging peak. Initial tunneling parameters: $V_S = 1$ V, $I_t = 15$ pA, $V_{AC} = 12$ mV (anchored), $V_S = 1$ V, $I_t = 3$ pA, $V_{AC} = 12$ mV (unanchored).

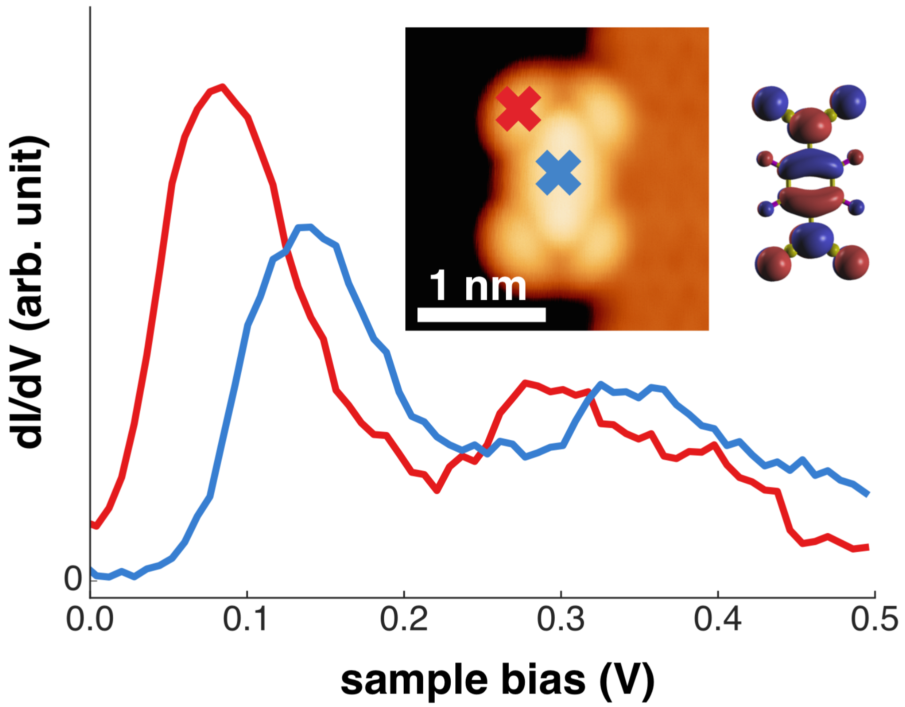

Supplementary Figure 2. The on-molecule dI/dV spectrum can vary depending on tip position (a known effect).[5] At the center of the molecule the p-like orbital (see theoretical orbital inset) occurs at a higher energy (blue dots) than at the more s-like orbital at the corner of the molecule (red dots). This is due to inelastic phonon-assisted tunneling[5] from the s-like orbitals of the tip to the p-like orbital at the molecule's center, leading to a peak energy higher than the true LUMO energy measured at the molecule's edge. We therefore measured spectra at the s-like corner of the molecule to extract the reproducible, precise LUMO energy (initial tunneling parameters: I_t = 15 pA , V_S = 1 V, V_{AC} = 12 mV).

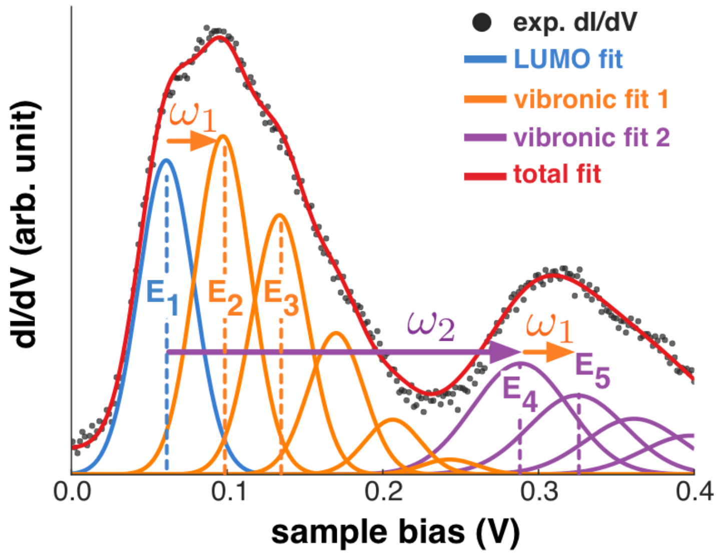

Supplementary Figure 3. Experimental dI/dV from Fig. 2 of main text, with a fit of evenly spaced Gaussians. The Gaussians of vibronic fit 1 are centered at energies $E_{\mathrm{L}} + n\,\omega_1$, where n=1,2,3,4,5, while the Gaussians of vibronic fit 2 are centered at energies $E_{\mathrm{L}} + \omega_2 + n\,\omega_1$, where with n=1,2,3. The best-fit values of the phonon energies are $\omega_1 \approx 37 \pm\ 7$ meV, $\omega_2 \approx 227 \pm\ 24$ meV. The total fit is the sum of all Gaussian fits (with a small constant offset).

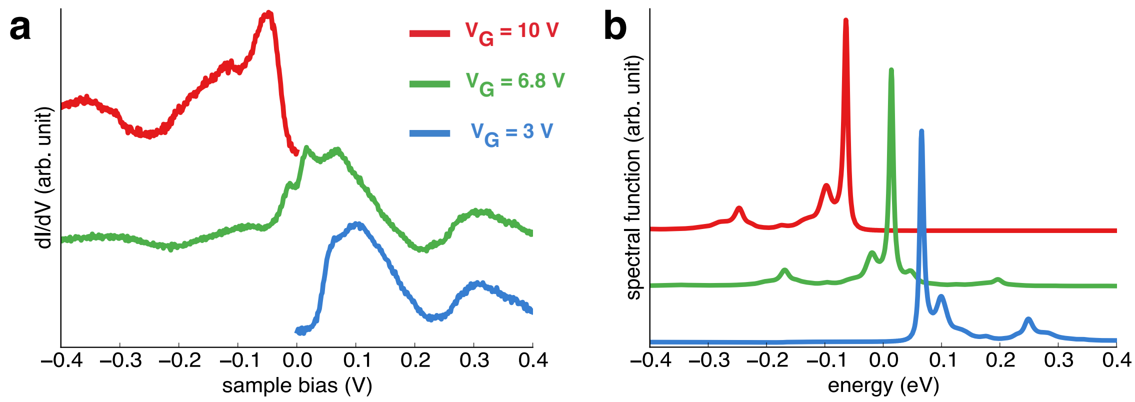

Supplementary Figure 4. Comparison of ab *initio* spectral function of F_4TCNQ LUMO state and experimental dI/dV for various fill states. **(a)** dI/dV for various gate voltages when the LUMO is completely filled, partially filled and empty (initial tunneling parameters: I_t = 30 pA , V_{S} = 0.4 V, V_{AC} = 8 mV (for V_{G} = 3 V, 6.8 V) and I_{t} = -40 pA , V_{S} = -0.7 V, V_{AC} = 8 mV (for V_{G} = 10 V)). The dI/dV for red and blue traces are featureless above and below E_{F}, respectively (not shown). **(b)** The theoretical spectral function when LUMO is completely filled, partially filled and empty. In both cases the vibronic satellites, including their relative energies above and below the LUMO are in qualitative agreement with experiment.

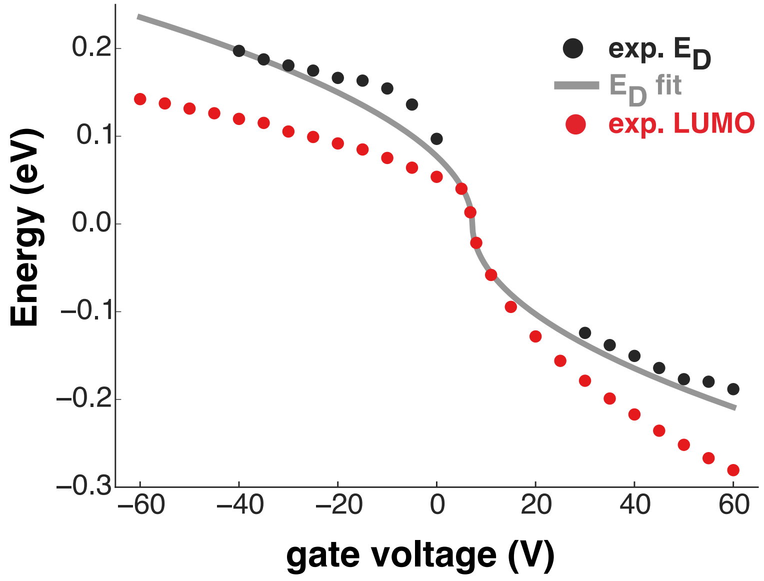

Supplementary Figure 5. Dirac point energy fit. For each gate voltage we measured the energy of the local minimum in a dI/dV spectrum on graphene as in Fig. 3b of the main text. We then subtracted 63 meV from the absolute values of those energies to account for the inelastic tunneling gap in the graphene spectrum.[4] The resulting energies are plotted as black dots. We then fit the parameter α in the function $E_D = \alpha \, \mathrm{sign}(V_g - V_g^0)\sqrt{|V_g - V_g^0|}$ to those data points (where we choose V_g^0 to be the gate voltage where the LUMO energy (red dots) has the steepest slope) because the local minimum at the Dirac point is difficult to determine close to charge neutrality due to the inelastic tunneling channel.[4]

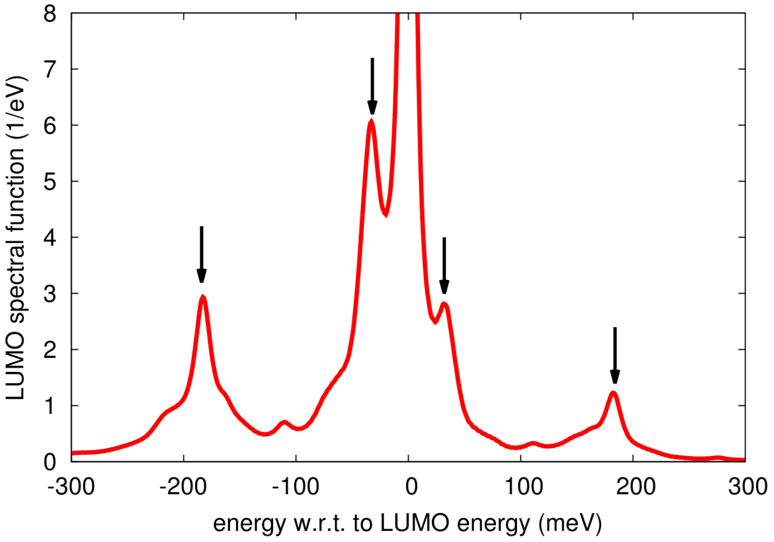

Supplementary Figure 6. *Ab initio* spectral function of F_4TCNQ LUMO state on ungated graphene. The LUMO is occupied by 0.7 electrons. Arrows indicate the positions of the phonon satellites. These phonon satellites are both above and below the Fermi energy because the LUMO is partially filled.

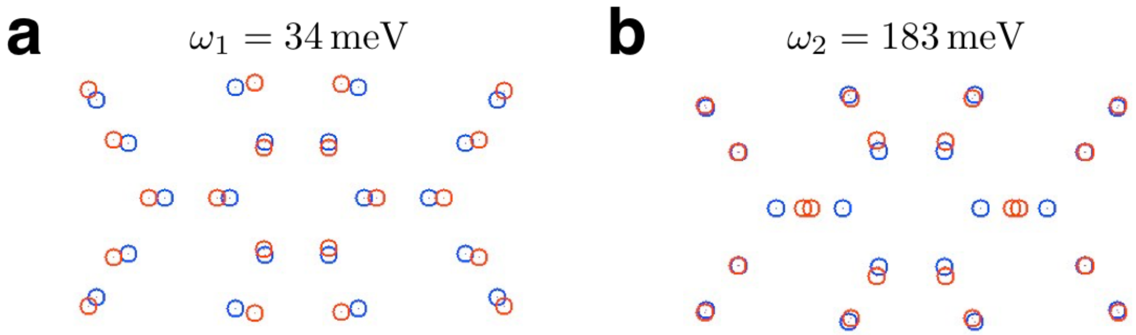

Supplementary Figure 7. Atomic displacement of the two phonons with the highest electron-phonon coupling. Both phonons exhibit A_g symmetry.

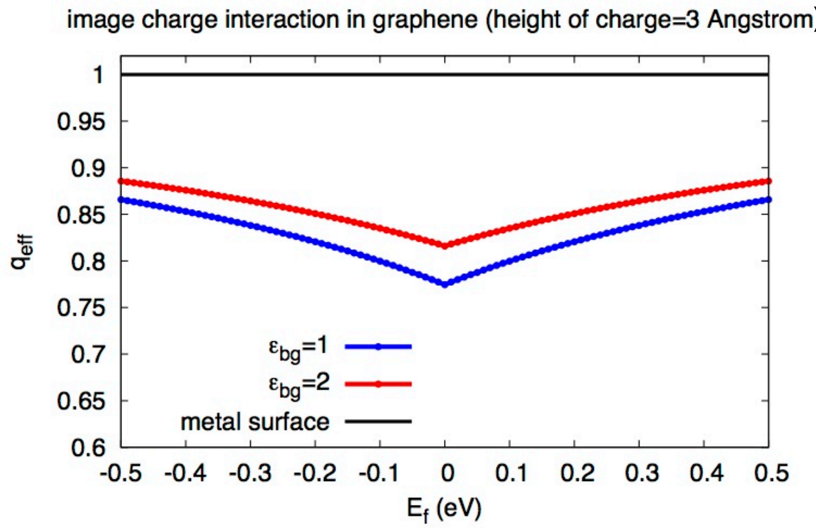

Supplementary Figure 8. The graphene image charge as function of the Fermi level for different substrates induced by a negative point charge located 3 Angstrom above the graphene plane. The blue curve shows the image charge for graphene in isolation ($\epsilon_{\mathrm{bg}} = 1$) and the red curve shows the result for graphene on BN. For comparison, we also show the image charge of a metallic substrate (black line).

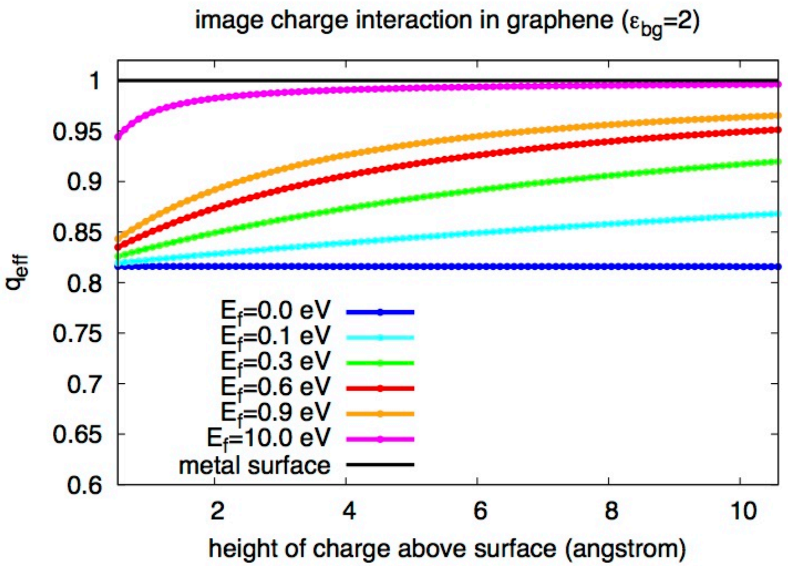

Supplementary Figure 9. The graphene image charge induced by a negative point charge above the graphene plane. The image charge increases for larger values of the Fermi energy, and also with the height of the charge above the graphene.

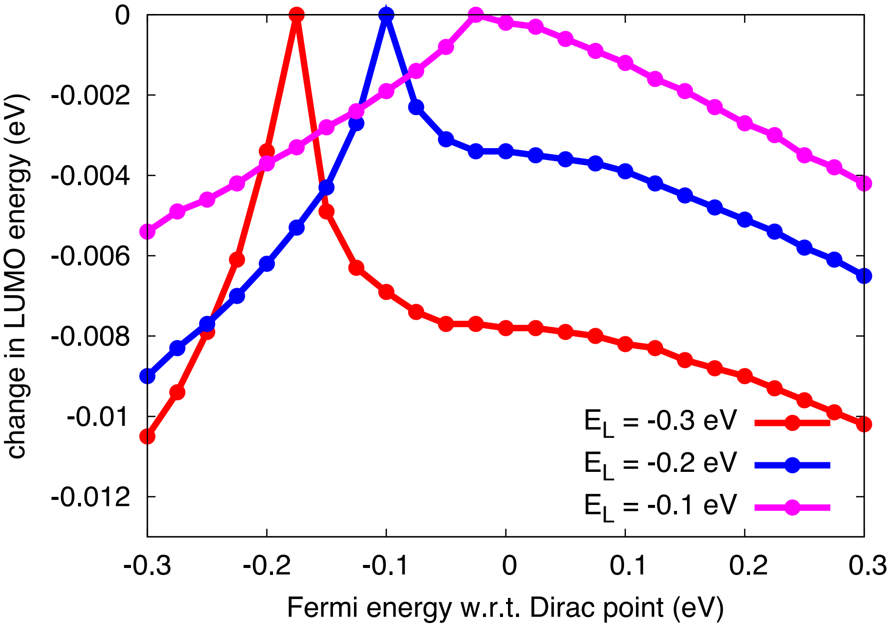

Supplementary Figure 10. Change in the F_4TCNQ LUMO energy due to intra-molecular electron-electron interactions. The calculated renormalization of the LUMO is significantly smaller than the effect of image charge interactions between the molecule and graphene.

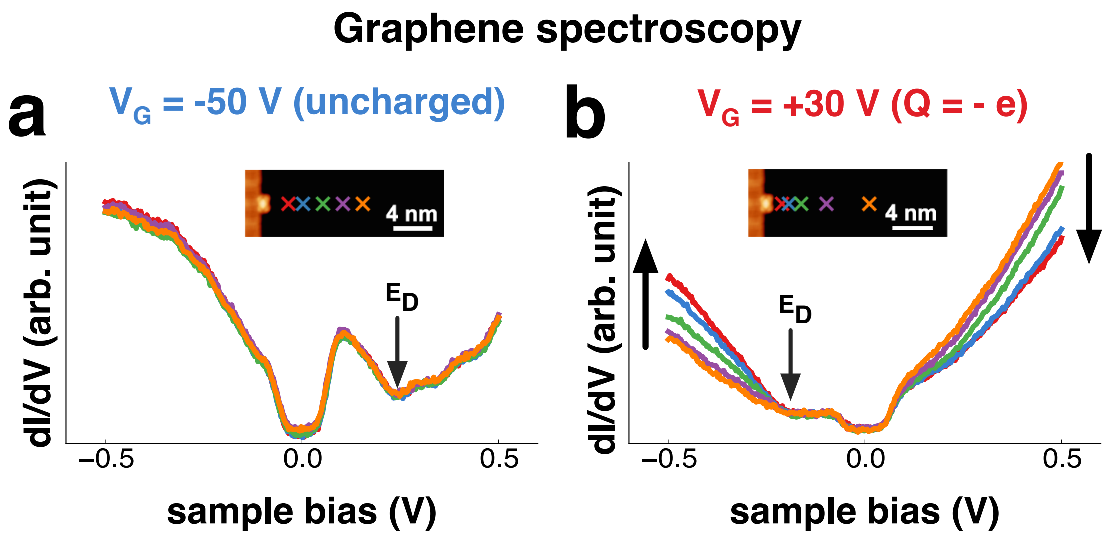

Supplementary Figure 11. Graphene spectroscopy near a single molecule reveals the molecule charge state. **(a)** d*I*/d*V* on graphene near an F_4TCNQ molecule at V_G = -50 V shows no distance dependence, indicating that the molecule is uncharged.[10] **(b)** For V_G = +30 V, d*I*/d*V* spectra on graphene show an asymmetry developing as the tip moves close to the molecule with d*I*/d*V* intensity decreasing above E_D and increasing below E_D. This is an indication of charge located at the molecule, which we determine to be $q \approx -e$ by comparison to previous results[10] (initial tunneling parameters: I_t = 50 pA, V_S = 0.5 V, V_{AC} = 12 mV at 10 nm away from F_4TCNQ).

Supplementary Notes
Supplementary Note 1. Electron-phonon coupling

We investigate the effects of electron-phonon interactions on tunneling spectra for STS measurements of F_4TCNQ molecules on graphene. The coupling of electrons to phonons renormalizes the electronic energies and gives rise to additional satellite features in the electron spectral function.

We carried out density-functional theory (DFT) calculations of the F_4TCNQ molecule on graphene using the QUANTUM ESPRESSO program package[1]. We employed the local density approximation, norm-conserving pseudopotentials, a plane-wave cutoff of 50 Ry and a 6×6 graphene supercell. The separation of periodic graphene sheets in the z-direction is 30 bohr. The Brillouin zone of the supercell was sampled with a 6×6×1 kpoint grid.

After determining the ground state geometry, we calculated the phonon modes and determined the electron-phonon coupling strengths using EPW computer code[2]. Electron energies ϵ_m, phonon frequencies ω_ν and electron-phonon couplings $g_{nm\nu}$ were used to evaluate the self-energy of an electron in state n due to electron-phonon interactions:

$$\Sigma_n^{ep}(\omega) = \sum_{m\nu} \left|g_{nm\mu}\right|^2 \left[\frac{f_m}{\omega-\epsilon_m+\omega_\nu+i\eta} + \frac{1-f_m}{\omega-\epsilon_m-\omega_\nu+i\eta}\right],$$

where f_m denotes the occupancy of state m (given by the Fermi distribution) and we have assumed $T = 0$ K and set the phonon occupancies to zero. We have also found that to a good approximation the sum over m can be reduced to keeping only the $m = n$ contributions.

Then, the electron spectral function $A_n(\omega) = \frac{1}{\pi} \times |\mathrm{Im}\, G_n(\omega)|$ (where $G_n(\omega) = \int dt \exp(i\omega t)\, G_n(t)$ denotes the electron Green's function) is computed using the generalized cumulant expansion approach of Story, Kas, Vila, Rehr and Verstraete[3].

$$G_n(t) = -ie^{-i\epsilon_n t}\, e^{C_n(t)}\, \Theta(t)$$

$$C_n(t) = \int_{-\infty}^{\infty} d\omega\, \beta_n(\omega)\, \frac{e^{i\omega t} - i\omega t - 1}{\omega^2}$$

$$\beta_n(\omega) = \frac{1}{\pi}\left|\mathrm{Im}\, \Sigma_n^{ep}(\epsilon_n - \omega)\right|,$$

where C_n denotes the cumulant function.

For the molecule, the imaginary part of the self-energy is a sum of delta-functions

$$\left|\mathrm{Im}\, \Sigma_n^{ep}(\omega)\right| = \pi \sum_\nu |g_{nn\nu}|^2 [(1-f_n)\, \delta(\omega-\epsilon_n-\omega_\nu) + f_n\, \delta(\omega-\epsilon_n+\omega_\nu)]$$

$$\beta_n(\omega) = \sum_\nu |g_{nn\nu}|^2 [(1-f_n)\, \delta(\omega+\omega_\nu) + f_n \delta(\omega-\omega_\nu)]$$

$$C_n^{sat}(t) = \sum_\nu \frac{|g_{nn\nu}|^2}{\omega_\nu^2} \left[(1-f_n)\, e^{-i\omega_\nu t} + f_n e^{i\omega_\nu t}\right]$$

$$C_n^{qp}(t) = -\sum_\nu \frac{|g_{nn\nu}|^2}{\omega_\nu^2} - it \sum_\nu \frac{|g_{nn\nu}|^2}{\omega_\nu} [2f_n - 1] \equiv -a_n - i\Delta E_n t,$$

where we separated the cumulant function into a quasiparticle contribution C_n^{qp} and a satellite contribution C_n^{sat}.

Plugging everything into the expression for the spectral function and Taylor expanding in C_n^{sat} yields

$$A_n(\omega) = Z\left[\delta(\omega - E_n) + \sum_\nu \frac{|g_{nn\nu}|^2}{\omega_\nu^2}\{(1 - f_n)\,\delta(\omega - E_n - \omega_\nu) + f_n\,\delta(\omega - E_n + \omega_\nu)\} + \cdots\right].$$

Supplementary Figure 6 shows the corresponding spectral function of the LUMO state. When the molecule is placed on graphene, approximately 0.7 electrons are transferred into the LUMO. As a consequence of this partial occupancy, phonon satellites occur on both sides of the LUMO quasiparticle peak. On each side, we observe two prominent satellite features: one at ~30 meV from the quasiparticle peak, originating from a uniaxial stretching mode with A_g symmetry, and another feature at ~180 meV, originating from a breathing mode of the inner carbon ring with A_g symmetry (Supplementary Figure 7).

We measured the experimental phonon energies ω_1 and ω_2 by fitting the dI/dV spectrum of the molecule at $V_G = 0$ V with multiple Gaussians (Supplementary Figure 3). In our fitting procedure we fit the first main peak separately from the second main peak. Fitting the first main peak with six evenly spaced Gaussians yielded ω_1. Fitting the second main peak with four evenly spaced Gaussians (separated by ω_1) yielded $\omega_2 = E_4 - E_1$.

Supplementary Note 2. Image Charge Effect.

In this section we explain the renormalization of the F_4TCNQ LUMO energy due to the presence of the graphene substrate. In contrast to typical bulk substrates graphene can be doped via gating, leading to a gate-dependent image charge effect.

The renormalization of molecular levels due to image charge corrections caused by the presence of a (semi-infinite) substrate has been investigated by Neaton, Hybertsen and Louie[6]. These authors find that the correction of the quasiparticle levels of a molecule is given by

$$\Delta E_{QP} = -\frac{1}{2}\delta U \text{ , for a completely filled level}$$
$$\Delta E_{QP} = +\frac{1}{2}\delta U \text{ , for an empty level,}$$

with $\delta U = \langle\psi_M^2|\Delta W|\psi_M^2\rangle$ (ΔW denotes the change in the screened Coulomb interaction due to the presence of the substrate and ψ_M denotes the wave function of the molecular orbital). Typically, the presence of a substrate increases the screening and thus δU is *negative*.

Before we apply this theory to F_4TCNQ on graphene, we discuss the image charge correction for a molecular level that is occupied by a single electron. The theory of Neaton, Hybertsen and Louie can be generalized to this case by noting that the energy needed to remove an electron from this system, i.e. the ionization potential (IP), equals the energy required to add an electron to the molecule with an empty level, i.e. its electron affinity (EA), because

$$E_{QP}^{IP}(N = 1) = E^N - E^{N-1} = E^{\tilde{N}+1} - E^{\tilde{N}} = E_{QP}^{EA}(\tilde{N} = 0).$$

The energy renormalization of the empty level E_{QP}^{EA}, however, is given by the theory of Neaton, Hybertsen and Louie [note that $E_{QP}^{IP}(N - 1) = E_{QP}^{EA}(\tilde{N} = 0)$ also without the substrate and

therefore the substrate-induced renormalization must be the same for $E_{QP}^{IP}(N = 1)$ and $E_{QP}^{EA}(\tilde{N} = 0)$].

We illustrate the result of the previous paragraph for the case of F_4TCNQ on doped graphene. In the neutral F_4TCNQ molecule the LUMO is completely empty. We assume that the presence of an extra electron in the LUMO of the molecule (assumed to be located a distance z^* above the graphene plane) gives rise to an extra contribution to the total energy of $-|e|Q(\epsilon_F)/(2z^*)$, where $|e|$ denotes the absolute magnitude of the electron charge and $Q(\epsilon_F) > 0$ denotes the doping-dependent image charge induced in the graphene.

For negative gate voltages, the LUMO is empty. The cost of transferring an electron from the tip into the empty LUMO is given by

$$E(add\ electron) = E_{tot}(1\ el, \epsilon_F) - E_{tot}(0\ el, \epsilon_F) - \epsilon_F,$$

where $E_{tot}(1\ el, \epsilon_F)$ and $E_{tot}(0\ el, \epsilon_F)$ denote the total energy of the molecule plus graphene system with a singly occupied or an empty LUMO, respectively. We can approximate these total energies as

$$E_{tot}(0\ el, \epsilon_F) = E_{gra}(\epsilon_F) + E_{mol}(0\ el, \epsilon_F)$$

and

$$E_{tot}(1\ el, \epsilon_F) = E_{gra}(\epsilon_F) + E_{mol}(1\ el, \epsilon_F) - \frac{|e|Q(\epsilon_F)}{2z^*},$$

where E_{gra} denotes the energy of graphene without the molecule and E_{mol} denotes the energy of the molecule without the graphene. Inserting this into the equation for $E(add\ electron)$ yields

$$E(add\ electron) = E_{mol}(1\ el, \epsilon_F) - E_{mol}(0\ el, \epsilon_F) - \frac{|e|Q(\epsilon_F)}{2z^*} - \epsilon_F.$$

This demonstrates that the image charge interaction reduces the energy of transferring an electron from the tip to the molecule.

For positive gate voltages, the LUMO is occupied by one electron and the energy for transferring this electron from the LUMO to the tip is given by

$$\begin{aligned} E(remove\ electron) &= E_{tot}(0\ el, \epsilon_F) - E_{tot}(1\ el, \epsilon_F) + \epsilon_F \\ &= E_{mol}(0\ el, \epsilon_F) - E_{mol}(1\ el, \epsilon_F) + \frac{|e|Q(\epsilon_F)}{2z^*} + \epsilon_F, \end{aligned}$$

indicating that the image charge interaction increases the energy needed to remove the electron from the LUMO.

To calculate the size of the induced image charge in doped graphene, we model the extra electron in the LUMO level as a point charge located a distance z^* above the graphene plane (which is assumed to be perfectly two-dimensional) and find for the Fermi-level dependent quasiparticle energy

$$\begin{aligned} E_{QP}(\epsilon_F) - E_{QP}(\epsilon_F = 0) &= -\frac{1}{2}[\delta U(\epsilon_F) - \delta U(0)] \\ &= -\frac{1}{2}\int_0^\infty dq\, \{\epsilon_q^{-1}(|\epsilon_F|) - \epsilon_q^{-1}(0)\} e^{-2qz^*}, \end{aligned}$$

where ϵ_q denotes the graphene dielectric function, which depends only on the absolute magnitude of the Fermi energy. As a consequence, the LUMO level renormalization is symmetric around the Dirac point $\epsilon_F = 0$. Using the long wavelength limit of the dielectric

function of graphene (see below), a simple scaling argument shows that $\Delta E_{QP}(\epsilon_F) = \epsilon_F\, F(k_F z^*)$ with k_F denoting the Fermi momentum. For small doping $k_F z^* \ll 1$ and $\Delta E_{QP}(\epsilon_F) \approx \epsilon_F\, F(0)$. Details of the calculation are found in the next section.

Supplementary Note 3. A simple model for the graphene image charge correction

In this section, we compute the renormalization of the molecule level due to the presence of the graphene below the molecule. In particular, we consider adding an extra electron to the molecule (located at position $[0\ 0\ z^*]$ above the graphene) and compute its interaction energy with the field that is induced in the graphene substrate.

The extra electron creates a field $\phi_{ext}(\boldsymbol{r})$ that induces a charge density $\delta n(\boldsymbol{r}) = \delta n(x,y)\,\delta(z)$ in the graphene plane.

The resulting potential due to δn is given by

$$\phi_{ind}(\boldsymbol{r}) = \int d^3r'\, v(\boldsymbol{r}-\boldsymbol{r}')\,\delta n(\boldsymbol{r}')$$

Within linear response theory we can compute δn *via*

$$\delta n(x,y) = \int dx'dy'\,\chi(x-x',y-y')\,\phi_{ext}(x',y')\,,$$

where $\phi_{ext}(x,y) = \frac{1}{\sqrt{x^2+y^2+z^{*2}}}$ and $\chi(q)$ denotes the interacting charge susceptibility of graphene, which is related to the dielectric constant *via* $v(q)\,\chi(q) = \epsilon^{-1}(q) - 1$.

Next, we transform the above expression to Fourier space. For this we need

$$\int dx\, dy \frac{1}{\sqrt{x^2+y^2+z^{*2}}}\, e^{i(q_x x + q_y y)} = \frac{2\pi}{|q|} e^{-|q|z^*},$$

with $q = \sqrt{q_x^2 + q_y^2}$.

We can now calculate $\delta n(x,y) = \delta n(r)$ according to

$$\delta n(r) = \int \frac{d^2q}{(2\pi)^2}\chi(q)\frac{2\pi}{q}e^{-qz^*}e^{-i\boldsymbol{q}\boldsymbol{r}} = \int_0^\infty dq\ \chi(q) J_0(qr) e^{-qz^*}\,,$$

where we introduced the Bessel function of the first kind J_0.

Finally, we can evaluate the induced potential at the position of the extra electron on the molecule

$$\delta U = \phi_{ind}(\boldsymbol{r} = [0,0,z^*]) = \int \frac{dx\, dy}{(2\pi)^2} \frac{1}{\sqrt{x^2+y^2+z^{*2}}}\,\delta n(x,y)$$

$$= \int_0^\infty dq\,[\epsilon^{-1}(q) - 1]\, e^{-2qz^*}.$$

To evaluate this expression, an approximation for the static dielectric function of graphene is needed. Here we use the result of Stauber et al.[7]

$$\epsilon(q) = \epsilon_{bg} - v(q)\,\chi^0(q)$$

$$\chi^0(q) = -\frac{2k_F}{\pi v_F} + \Theta(q - 2k_F)\frac{q}{2\pi v_F}\, G\left(\frac{2k_F}{q}\right)$$

$$G(x) = x\sqrt{1-x^2} - \mathrm{acos}(x),$$

where ϵ_{bg} denotes a background dielectric constant, k_F is the Fermi wave vector and v_F the Fermi velocity.

Two limiting cases can be studied analytically: i) intrinsic graphene, i.e. $k_F = 0$, and ii) heavily doped graphene. For intrinsic graphene, we find $\chi^0(q) = -q/(4v_F)$ and the induced potential is

$$\delta U = \frac{q_{eff}}{2z^*},$$

with $q_{eff} = \frac{2v_F}{\pi + 2v_F\epsilon_{bg}} - 1$. Note that the induced potential looks exactly like the image charge potential for a point charge over the surface of an insulator with bulk dielectric constant $\epsilon = (1 - q_{eff})/(1 + q_{eff})$. We thus find that the image charge induced by graphene in isolation (i.e. $\epsilon_{bg} = 1$) is the same as that for an insulator with bulk dielectric constant $\epsilon = 7.9$. The second case we study is heavily doped graphene. In this case, $\chi^0(q) = -\frac{2k_F}{\pi v_F}$ and $\delta U = -\frac{1}{2z^*}$, which is the same image charge response as for a bulk metal surface.

Supplementary Note 4. Intra-molecular electron-electron interactions

In addition to electron-electron interactions between the molecule and the graphene substrate, we also investigated the effect of electron-electron interactions within the molecule and show that for the case of F_4TCNQ on doped graphene the effect of these interactions is much smaller than the effect of the image charge interactions between the molecule and graphene.

We use the Anderson model to describe intra-molecular electron-electron interactions. Specifically, the Hamiltonian is given by

$$H = H_{gra} + H_{mol} + H_{hyb},$$

where H_{gra} denotes the graphene-tight-binding Hamiltonian and $H_{mol} = E_L n_L + U n_{L\uparrow} n_{L\downarrow}$ denotes the Hamiltonian of the isolated molecule (with the LUMO electron number operator $n_L = n_{L\uparrow} + n_{L\downarrow}$ and E_L and U denote the LUMO energy level and the onsite Coulomb energy, respectively). Finally, the hybridization term H_{hyb} describes the hopping of electrons from the graphene onto the molecule and is proportional to the hybridization energy V.[8]

We now describe how we determine U, V, and E_{L} for F_4TCNQ on graphene. We first carried out a DFT calculation of F_4TCNQ on graphene and determine $V = 1.0\ eV$ from the width of the LUMO resonance in the density of states. For the isolated F_4TCNQ molecule, we obtain $U = 3.1\ eV$ from the spin splitting of the singly occupied LUMO level, which we calculate using the GW method. Determining E_{L} (with respect to the Dirac point energy) requires, in principle, a GW calculation of F_4TCNQ on graphene, which is computationally very challenging. Experimentally, E_{L} is found to be slightly below the Dirac point energy and we therefore carry out calculations for $E_{\mathrm{L}} = -0.3\ eV$, $-0.2\ eV$ and $-0.1\ eV$.

In the presence of large on-site interactions, U, the Anderson model is known to exhibit Kondo physics. For the parameters at hand, however, a simple estimate shows that the corresponding energy scale - the Kondo temperature - is at least one order of magnitude smaller than the temperature at which the experiment is carried out. Hence, our physical configuration is not governed by Kondo physics but by charging effects, which are much simpler to describe theoretically. Since $U = 3.1\ eV$ is much larger than the hydridization energy $V = 1.0\ eV$, we employ the Non-Crossing Approximation to determine properties of the Anderson model[9]. This approach is non-perturbative in U, but treats the hybridization V as a small parameter. In practice, we first evaluate the corrections due to the finite value of V for the energies of the states with zero and one electron in the LUMO (for $V = 0$, these are given by $E_0 = 0$ and $E_1 = E_{\mathrm{L}} - \epsilon_{\mathrm{F}}$). In a second step, we determine the peak in the tunneling spectrum by computing the energy difference $E_1 - E_0$.

Supplementary Figure 10 shows the results of our calculations for the change in the LUMO energy due to intra-molecular electron-electron interactions as a function of the graphene doping. We find that the LUMO renormalization for the experimentally relevant range of doping levels is at most 10 meV and thus significantly smaller than the renormalization due to image charge effects.

Supplementary References

1 Paolo, G. *et al.* QUANTUM ESPRESSO: a modular and open-source software project for quantum simulations of materials. *Journal of Physics: Condensed Matter* **21**, 395502 (2009).

2 Noffsinger, J. *et al.* EPW: A program for calculating the electron–phonon coupling using maximally localized Wannier functions. *Computer Physics Communications* **181**, 2140-2148, doi:http://dx.doi.org/10.1016/j.cpc.2010.08.027 (2010).

3 Story, S. M., Kas, J. J., Vila, F. D., Verstraete, M. J. & Rehr, J. J. Cumulant expansion for phonon contributions to the electron spectral function. *Physical Review B* **90**, 195135 (2014).

4 Zhang, Y. *et al.* Giant phonon-induced conductance in scanning tunnelling spectroscopy of gate-tunable graphene. *Nat Phys* **4**, 627-630, doi:http://www.nature.com/nphys/journal/v4/n8/suppinfo/nphys1022_S1.html (2008).

5 Pavliček, N., Swart, I., Niedenführ, J., Meyer, G. & Repp, J. Symmetry Dependence of Vibration-Assisted Tunneling. *Physical Review Letters* **110**, 136101 (2013).

6 Neaton, J. B., Hybertsen, M. S. & Louie, S. G. Renormalization of Molecular Electronic Levels at Metal-Molecule Interfaces. *Physical Review Letters* **97**, 216405 (2006).

7 Wunsch, B., Stauber, T., Sols, F. & Guinea, F. Dynamical polarization of graphene at finite doping. *New Journal of Physics* **8**, 318 (2006).

8 Uchoa, B., Kotov, V. N., Peres, N. M. R. & Castro Neto, A. H. Localized Magnetic States in Graphene. *Physical Review Letters* **101**, 026805 (2008).

9 Grewe, N. SPECTRAL PROPERTIES OF THE ANDERSON MODEL. *Zeitschrift Fur Physik B-Condensed Matter* **53**, 271-282, doi:10.1007/bf01436289 (1983).

10 Wang, Y. *et al.* Mapping Dirac quasiparticles near a single Coulomb impurity on graphene. *Nat Phys* **8**, 653-657, doi:http://www.nature.com/nphys/journal/v8/n9/abs/nphys2379.html#supplementary-information (2012).
